# Association Between Partner Phubbing and Romantic Relationship Satisfaction in a Sample of Portuguese Adults

**DOI:** 10.1192/j.eurpsy.2026.10859

**Published:** 2026-07-31

**Authors:** N. Jordão, B. R. Maia

**Affiliations:** 1 Faculty of Philosophy and Social Sciences; 2 Faculty of Philosophy and Social Sciences, Centre for Philosophical and Humanistic Studies, Universidade Católica Portuguesa, Braga, Portugal

## Abstract

**Introduction:**

Partner phubbing refers to the act of ignoring a romantic partner in favor of one’s mobile phone thereby compromising romantic relationship satisfaction.

**Objectives:**

This study aims to explore the association between partner phubbing and romantic relationship satisfaction in Portuguese adults.

**Methods:**

A sample comprised of 70 participants with an average age of 46.85 years completed the Partner Phubbing Scale and the Dyadic Adjustment Scale.

**Results:**

The results showed that partner phubbing is negatively correlated with Cohesion (.-2**), Consensus (*r* = -.41**), and Dyadic Adjustment (*r* = -.38**). Significant differences were found in Dyadic Adjustment and in Consensus between adults/33 and 45 years old (*M* = 48.91, *SD* = 7.70; *M* = 23.41, *SD* = 3.66, respectively) and middle-age adults/46-59 years old (*M* = 53.17, *SD* = 8.53; *M* = 25.41, *SD* = 3.30, respectively), *t* (66) = -.241, *p* =.01; t (66) = -.2.36, *p* = .02, respectively; the effect size was moderate (.08 and .07, respectively). Significant differences were also found for relationship satisfaction between males (*M* = 16.17, *SD* = 3.96) and females (*M* = 14.17, *SD* = 3.34), *t* (68) = -2.89, *p* = .005, with a medium effect (.10).

**Conclusions:**

Therefore, our results highligh the negative relationship between partner phubbing and romantic relationship satisfaction and the need to develop psychosocial prevention and intervention strategies regarding partner phubbing.

**Disclosure of Interest:**

N. Jordão: None Declared, B. Maia Grant / Research support from: UIDB/00683/2020, FCT

